# Inflammatory markers as predictors of liver fibrosis in type 2 diabetes patients with metabolic dysfunction-associated fatty liver disease

**DOI:** 10.3389/fendo.2025.1556646

**Published:** 2025-04-08

**Authors:** Yange Tang, Yulong Deng, Gengliang Zhang, Yanjun Wang, Jing Wang, Jie Wu, Mengjin Gu

**Affiliations:** ^1^ Department of Endocrinology, Hebei Traditional Chinese Medicine Hospital, Shijiazhuang, China; ^2^ Department of Orthopaedics and Traumatology, Hebei Traditional Chinese Medicine Hospital, Shijiazhuang, China; ^3^ Department of Anesthesiology, Zhengding County People’s Hospital, Shijiazhuang, China

**Keywords:** inflammatory markers, type 2 diabetes mellitus, metabolic dysfunction-associated fatty liver disease, liver fibrosis, diagnosis

## Abstract

**Objective:**

This study investigates the link between inflammatory markers and liver fibrosis in type 2 diabetes mellitus (T2DM) patients with metabolic dysfunction-associated fatty liver disease (MAFLD).

**Methods:**

From Oct 2020 to Oct 2024, 769 hospitalized T2DM patients were studied. They were split into Control (n=389) and Experimental groups (T2DM with MAFLD, n=380). The Experimental group was further divided based on FIB-4 scores into non-fibrosis (FIB-4< 1.3, n=267), suspected fibrosis (1.3 ≤ FIB-4 ≤ 2.67, n=99), and advanced fibrosis (FIB-4 > 2.67, n=14). Logistic regression identified factors affecting liver fibrosis, while ROC analysis assessed the predictive value of NLR, SIRI, PLR, and PHR for liver fibrosis in T2DM-MAFLD patients.

**Results:**

The Experimental group showed higher BMI, FPG, TG, TC, LDL-C, ALT, AST, ALB, GGT, and SUA, but lower age, diabetes duration, MPV, and HDL-C (P< 0.05). Compared to non-fibrosis, suspected fibrosis had higher age, diabetes duration, MPV, AST, and NLR, and lower LY, PLR, PHR. Advanced fibrosis featured higher age, AST, NLR, FPG, HbA1c, SIRI, and lower LY, RBC, LDL-C, PLR, PHR, Hb, PLT, and ALB (P< 0.05). Logistic regression identified NLR, SIRI, PLR, and PHR as significant factors for liver fibrosis. ROC analysis showed AUCs of 0.712 (NLR), 0.757 (SIRI), 0.703 (PLR), and 0.806 (PHR) with sensitivities and specificities varying among markers. Optimal cut-offs were 1.573 (NLR), 1.465 (SIRI), 110.819 (PLR), and 185.379 (PHR).

**Conclusions:**

NLR, SIRI, PLR, and PHR significantly influence liver fibrosis in T2DM patients with MAFLD, aiding in its diagnosis and management.

## 1 Introduction

The liver is a central organ responsible for regulating metabolic homeostasis in the body. As such, it plays a pivotal role in maintaining glucose, lipid, and protein balance. Among various liver conditions, metabolic dysfunction-associated fatty liver disease (MAFLD) is emerging as a global health concern. The term “non-alcoholic fatty liver disease” (NAFLD) was officially redefined as MAFLD in 2020 to reflect a broader understanding of its pathophysiology, which includes metabolic disturbances beyond just fat accumulation in the liver (1). This reclassification was accompanied by the release of international expert consensus statements and updated diagnostic criteria, which provide a more accurate framework for the identification and management of MAFLD (2). These changes in terminology and diagnostic criteria have facilitated better patient stratification and understanding of the disease’s underlying pathogenesis.

MAFLD, if left untreated, can progress to liver fibrosis, cirrhosis, and ultimately hepatocellular carcinoma, significantly increasing liver-related morbidity and mortality. The progression from steatosis to fibrosis is closely linked to chronic inflammation, characterized by immune cell infiltration, cytokine release, and activation of hepatic stellate cells, which promote extracellular matrix deposition (3). Although liver biopsy remains the gold standard for diagnosing liver fibrosis, it is invasive and carries certain risks, such as infection and bleeding, making it less suitable for widespread use, especially in patients with early-stage fibrosis. Consequently, non-invasive methods such as the FIB-4 score have become widely adopted for assessing liver fibrosis severity in clinical practice (4). The FIB-4 score, based on a combination of age, liver enzymes (AST and ALT), and platelet count, allows for stratifying patients with fatty liver disease based on the likelihood of having significant fibrosis.

The prevalence of both type 2 diabetes mellitus (T2DM) and MAFLD has been rising at alarming rates worldwide, leading to increased clinical attention towards the co-occurrence of these two metabolic disorders. T2DM is a well-established risk factor for the development of MAFLD, with the presence of insulin resistance and dysregulated lipid metabolism playing central roles in its pathogenesis (5). Furthermore, inflammation is thought to play a significant role in the progression of MAFLD to more severe liver damage, including fibrosis and cirrhosis. Chronic low-grade inflammation, driven by adipose tissue dysfunction, oxidative stress, and gut microbiota dysbiosis, creates a pro-fibrogenic microenvironment in the liver (6). This inflammatory milieu is reflected in systemic biomarkers, which have emerged as promising tools for non-invasive risk stratification.

Inflammatory markers such as the neutrophil-to-lymphocyte ratio (NLR), systemic inflammation response index (SIRI), platelet-to-lymphocyte ratio (PLR), and platelet-to-high-density lipoprotein cholesterol ratio (PHR) are gaining prominence in metabolic and hepatic diseases. NLR, calculated as the ratio of neutrophils to lymphocytes, reflects systemic inflammation and immune imbalance (7, 8). Elevated NLR has been associated with severity of liver fibrosis in MAFLD and predicts adverse outcomes in cardiovascular diseases (9, 10). SIRI, integrating neutrophil, monocyte, and lymphocyte counts, is a novel marker of systemic inflammation (11). Recent studies highlight its predictive value for cardiovascular events (12) and hepatic complications (13) in metabolic syndrome. PLR, representing the balance between thrombotic and inflammatory pathways, has shown inverse correlations with MAFLD severity and insulin resistance (14). PHR, a composite marker of platelet activity and lipid metabolism, is positively correlated with hepatic steatosis and fibrosis progression, offering insights into the interplay between dyslipidemia and inflammation (15). These indices are cost-effective, readily available from routine blood tests, and provide a holistic view of the inflammatory state, making them attractive for clinical application.

This study seeks to examine the correlation between these inflammatory markers (NLR, SIRI, PLR, and PHR) and liver fibrosis in patients with T2DM and MAFLD, with the goal of providing a clinical basis for early diagnosis and targeted intervention. By evaluating the role of these inflammatory indices in liver fibrosis, we hope to enhance the understanding of the complex relationship between systemic inflammation, metabolic disorders, and liver disease progression, which could potentially inform future therapeutic strategies.

## 2 Subjects and methods

### 2.1 Study subjects

A total of 769 patients with T2DM who were hospitalized at our institution from October 2020 to October 2024 were included in the study. All participants met the diagnostic criteria for diabetes as outlined by the World Health Organization (WHO) in 1999 (16). The diagnosis of MAFLD was established according to the “Guidelines for the Prevention and Treatment of Metabolic-Associated (Non-Alcoholic) Fatty Liver Disease (2024 Edition)” (17), which requires the presence of hepatic steatosis (detected via abdominal ultrasound or computed tomography [CT]) combined with at least one of the following metabolic risk abnormalities: (1) overweight/obesity (BMI ≥23 kg/m²), (2) type 2 diabetes mellitus, or (3) evidence of metabolic dysregulation (elevated blood pressure, triglycerides ≥150 mg/dL, HDL-C<40 mg/dL in males or<50 mg/dL in females, or prediabetes) (2).

Exclusion criteria included: patients with type 1 diabetes mellitus (T1DM), gestational diabetes mellitus (GDM), other specific types of diabetes, or those with acute severe diabetes complications; individuals with severe liver or kidney dysfunction, long-term heavy alcohol consumption, viral hepatitis, autoimmune hepatitis, or drug-induced liver injury; patients with hepatic hemangioma, intrahepatic calcification, or those who had undergone liver surgery that could affect liver function test results; individuals with acute or chronic infectious diseases, malignant tumors, or hematological disorders; and patients with incomplete clinical data.

Based on the presence or absence of MAFLD, patients were divided into two main groups: the Control group (n=389) and the Experimental group (n=380). The Experimental group was further stratified into three subgroups based on the FIB-4 score: non-fibrosis subgroup (FIB-4<1.3, n=267), suspected fibrosis subgroup (1.3 ≤ FIB-4 ≤ 2.67, n=99), and advanced fibrosis subgroup (FIB-4 >2.67, n=14) (18). This study was approved by the hospital’s ethics committee, and the requirement for informed consent was waived due to the retrospective nature of the study.

### 2.2 Research methods

Demographic data including gender, age, and diabetes duration were collected for all subjects. Height and weight were measured, and body mass index (BMI) was subsequently calculated. After an overnight fast of at least 8 hours, 5 mL of venous blood was drawn from the antecubital vein the following morning. Hematological parameters, including white blood cell count (WBC), neutrophil count (NE), lymphocyte count (LY), monocyte count (MC), red blood cell count (RBC), hemoglobin (Hb), platelet count (PLT), platelet distribution width (PDW), mean platelet volume (MPV), mean corpuscular volume (MCV), and mean corpuscular hemoglobin (MCH), were measured using a Sysmex XN-9000 hematology analyzer (Sysmex Corporation, Kobe, Japan) with associated reagents (Lot No. XN-CA21, Sysmex).

Biochemical parameters, including alanine aminotransferase (ALT), aspartate aminotransferase (AST), albumin (ALB), alkaline phosphatase (ALP), γ-glutamyl transferase (GGT), serum creatinine (Scr), serum uric acid (SUA), triglycerides (TG), total cholesterol (TC), high-density lipoprotein cholesterol (HDL-C), low-density lipoprotein cholesterol (LDL-C), fasting plasma glucose (FPG), and glycated hemoglobin (HbA1c), were measured using a Roche Cobas 8000 automated biochemical analyzer (Roche Diagnostics, Basel, Switzerland) with standardized reagents (Lot No. 06657522, Roche).

The inflammatory markers were calculated as follows:

- Neutrophil-to-lymphocyte ratio (NLR): NE/LY, validated as a marker of systemic inflammation in metabolic diseases (19).
- Systemic inflammation response index (SIRI): NE × MC/LY, a composite index predictive of hepatic fibrosis in MAFLD (20).
- Platelet-to-lymphocyte ratio (PLR): PLT/LY, associated with insulin resistance and liver injury (21).
- Platelet-to-high-density lipoprotein cholesterol ratio (PHR): PLT/HDL-C, reflecting dyslipidemia-driven inflammation (22).

The FIB-4 score (23) was calculated using the following formula: $\text{FIB}-4=\frac{Age\times AST}{PLT\times \sqrt{ALT}}$ .

Doppler ultrasound (GE Logiq E10, General Electric, USA) was used to measure left ventricular ejection fraction (LVEF). Additionally, abdominal imaging results from Doppler ultrasound (Philips EPIQ 7, Philips Healthcare, Netherlands) and CT scans (Siemens SOMATOM Definition Edge, Siemens Healthineers, Germany) were collected to assess liver morphology and function.

### 2.3 Statistical analysis

Statistical analysis was performed using SPSS 26.0 software. Non-normally distributed continuous data were expressed as median (interquartile range) [M (QL, QU)], and intergroup comparisons were conducted using non-parametric tests. Pairwise comparisons between groups were performed using the Kruskal-Wallis one-way ANOVA test. Logistic regression analysis was utilized to identify the factors influencing liver fibrosis in T2DM patients with MAFLD. The diagnostic value of inflammatory markers (NLR, SIRI, PLR, and PHR) in predicting liver fibrosis was assessed using receiver operating characteristic (ROC) curve analysis. A P-value of less than 0.05 was considered statistically significant.

## 3 Results

### 3.1 Comparison of general characteristics and biochemical indicators between the two groups

The Experimental group exhibited significantly higher levels of BMI, LY, RBC, Hb, FPG, TG, TC, LDL-C, AST, ALT, ALB, GGT, and SUA compared to the Control group (P< 0.05). In contrast, the Experimental group showed significantly lower levels of age, duration of diabetes, mean platelet volume (MPV), and high-density lipoprotein cholesterol (HDL-C) (P< 0.05) (**Table 1**).

**Table 1:** Comparison of general data and biochemical indexes between the two groups [M (Q_L_, Q_U_), n (%)].

Variable	Control	Experimental
N (M/F)	389 (228/161)	380 (239/141)
Age (Years)	58.00 (52.00,65.00)	56.00 (48.00,63.00)*
DM duration (Months)	84.00 (13.00,178.00)	40.00 (3.00,131.00)*
BMI (kg/m^2^)	23.70 (22.27,26.37)	26.47 (24.42,28.85)*
WBC (×10^9^/L)	6.03 (5.06,7.20)	6.23 (5.18,7.32)
NE (×10^9^/L)	3.39 (2.71,4.37)	3.42 (2.79,4.37)
LY (×10^9^/L)	1.84 (1.42,2.33)	2.04 (1.59,2.44)*
MC (×10^9^/L)	0.44 (0.35,0.55)	0.46 (0.36,0.58)
RBC (×10^12^/L)	4.51 (4.17,4.84)	4.69 (4.35,5.02)*
Hb (g/L)	139 (128,150)	145 (134,154)*
PLT (×10^9^/L)	206 (171,248)	217 (183,255)
PDW (fl)	13.0 (11.5,14.7)	12.8 (11.7,15.0)
MPV (fl)	10.6 (9.9,11.3)	10.4 (9.9,11.1)*
MCV (fl)	91.2 (88.4,93.9)	91.1 (88.7,94.4)
MCH (pg)	30.7 (29.5,31.7)	30.8 (29.8,31.8)
FPG (mmol/L)	8.28 (6.60,11.35)	8.90 (7.12,11.59*
HbA1c (%)	8.3 (6.9,10.2)	8.4 (7.1,10.1)
TG (mmol/L)	1.30 (0.94,1.85)	1.86 (1.29,2.84)*
TC (mmol/L)	4.61 (3.87,5.49)	5.00 (4.15,5.63)*
HDL-C (mmol/L)	1.18 (1.00,1.39)	1.11 (0.95,1.31)*
LDL-C (mmol/L)	2.71 (2.06,3.4)	3.01 (2.34,3.5)*
AST (U/L)	16 (13,19)	18 (14,25)*
ALT (U/L)	16 (12,23)	21 (16,34)*
ALB (g/L)	43.8 (40.7,45.9)	44.5 (41.7,46.8)*
ALP (U/L)	74 (62,92)	74 (64,92)
GGT (U/L)	22 (16,33)	32 (22,51)*
Scr (µmol/L)	64 (55,72)	63 (55,73)
SUA (µmol/L)	278.0 (230.0,329.0)	313.2 (261.0,365.3)*
LVEF (%)	58 (58,59)	58 (58,59)

Note: vs. Control group, *P<0.05.

M (QL, QU), Median (Lower Quartile, Upper Quartile); N (M/F), Number of participants (Male/Female); DM, Diabetes Mellitus; BMI, Body Mass Index; WBC, White Blood Cell count; NE, Neutrophil count; LY, Lymphocyte count; MC, Monocyte count; RBC, Red Blood Cell count; Hb, Hemoglobin; PLT, Platelet count; PDW, Platelet Distribution Width; MPV, Mean Platelet Volume; MCV, Mean Corpuscular Volume; MCH, Mean Corpuscular Hemoglobin; FPG, Fasting Plasma Glucose; HbA1c, Hemoglobin A1c; TG, Triglycerides; TC, Total Cholesterol; HDL-C, High-Density Lipoprotein Cholesterol; LDL-C, Low-Density Lipoprotein Cholesterol; AST, Aspartate Aminotransferase; ALT, Alanine Aminotransferase; ALB, Albumin; ALP, Alkaline Phosphatase; GGT, Gamma-Glutamyl Transferase; Scr, Serum Creatinine; SUA, Serum Uric Acid; LVEF, Left Ventricular Ejection Fraction.

### 3.2 Comparison of general characteristics and biochemical indicators among subgroups

When compared to the non-fibrosis subgroup, the suspected fibrosis subgroup demonstrated increased age, duration of diabetes, MCV, AST, and NLR (P< 0.05). In contrast, LY, PLT, and PHR were significantly decreased (P< 0.05). The advanced fibrosis subgroup exhibited further increases in age, AST, and NLR (P< 0.05), while LY, RBC, LDL-C, PLR, and PHR were significantly lower (P< 0.05). Additionally, compared to the suspected fibrosis subgroup, the advanced fibrosis subgroup had elevated levels of FPG, HbA1c, and SIRI (P< 0.05), while Hb, PLT, and ALB levels were significantly lower (P< 0.05) (**Table 2**).

**Table 2:** Comparison of general data and biochemical indexes among subgroups[M (Q_L_, Q_U_), n (%)].

Variable	Non-Liver Fibrosis	Suspected Liver Fibrosis	Advanced Stage of Liver Fibrosis
**N (M/F)**	267 (171/96)	99 (62/37)	14 (6/8)
**Age (Years)**	52.00 (45.00, 59.00)	60.00 (56.00, 65.00)*	68.00 (60.00, 70.00)*
**DM Duration (Months)**	31.00 (2.00, 121.00)	86.00 (13.00, 176.00)*	29.00 (0.00, 124.00)
**BMI (kg/m²)**	26.30 (24.54, 28.80)	26.81 (23.94, 29.10)	25.30 (24.00, 31.90)
**WBC (×10^9^/L)**	6.33 (5.42, 7.51)	6.00 (4.95, 6.97)	5.20 (4.51, 6.91)
**NE (×10^9^/L)**	3.40 (2.75, 4.37)	3.48 (2.80, 4.26)	3.52 (3.16, 5.80)
**LY (×10^9^/L)**	2.11 (1.74, 2.49)	1.83 (1.51, 2.35)*	1.60 (1.23, 2.00)*
**MC (×10^9^/L)**	0.46 (0.35, 0.59)	0.44 (0.35, 0.58)	0.56 (0.47, 0.68)
**RBC (×10^12^/L)**	4.70 (4.36, 5.06)	4.71 (4.36, 4.95)	4.35 (4.01, 4.63)*#
**Hb (g/L)**	145 (134, 154)	146 (136, 155)	135 (122, 144)*#
**PLT (×10^9^/L)**	231 (198, 266)	189 (164, 215)*	130 (108, 154)*#
**PDW (fl)**	12.7 (11.6, 14.6)	13.0 (11.8, 15.8)	14.2 (13.0, 16.2)
**MPV (fl)**	10.3 (9.8, 11.0)	10.5 (10.1, 11.2)	10.6 (10.2, 10.9)
**MCV (fl)**	90.4 (88.1, 94.0)	92.6 (89.4, 96.3)*	90.7 (89.0, 93.8)
**MCH (pg)**	30.6 (29.7, 31.7)	31.1 (29.9, 32.2)	30.5 (29.4, 31.4)
**FPG (mmol/L)**	8.88 (7.10, 11.35)	8.80 (7.10, 11.59)	13.53 (11.28, 16.66)*#
**HbA1c (%)**	8.4 (7.1, 10.1)	8.0 (6.9, 9.1)	10.4 (9.3, 12.3)*#
**TG (mmol/L)**	1.96 (1.35, 2.90)	1.61 (1.15, 2.45)	1.94 (1.29, 2.64)
**TC (mmol/L)**	5.07 (4.27, 5.74)	4.66 (3.75, 5.60)	4.21 (3.57, 5.19)
**HDL-C (mmol/L)**	1.10 (0.95, 1.29)	1.14 (0.96, 1.34)	1.00 (0.73, 1.29)
**LDL-C (mmol/L)**	3.10 (2.51, 3.57)	2.82 (2.05, 3.41)	2.53 (1.90, 2.86)*
**AST (U/L)**	16 (13, 21)	23 (18, 35)*	34 (24, 59)*
**ALT (U/L)**	20 (15, 30)	25 (16, 48)	27 (19, 54)
**ALB (g/L)**	44.9 (42.1, 46.8)	44.1 (41.7, 46.7)	41.3 (36.4, 44.2)*#
**ALP (U/L)**	75 (64, 92)	71 (59, 92)	84 (69, 96)
**NLR**	1.68 (1.27, 2.09)	1.77 (1.46, 2.47)*	2.06 (1.74, 3.07)*
**SIRI**	0.73 (0.54, 1.08)	0.84 (0.57, 1.16)	1.32 (0.89, 3.12)*#
**PLR**	113.64 (88.19, 138.32)	101.32 (79.53, 127.35)	84.09 (64.52, 106.94)*
**PHR**	212.39 (168.52, 255.66)	168.21 (133.33, 200.00)*	135.64 (109.89, 169.31)*
**GGT (U/L)**	30 (22, 49)	33 (21, 70)	35 (25, 120)
**Scr (µmol/L)**	63 (54, 72)	65 (58, 77)	64 (52, 77)
**SUA (µmol/L)**	313.0 (261.0, 359.9)	318.9 (260.0, 376.3)	321.6 (181.6, 368.2)
**LVEF (%)**	58 (57, 60)	58 (58, 59)	58 (58, 58)

Notes: vs. Non-liver fibrosis subgroup, *P<0.05;vs. Suspected liver fibrosis subgroup, #P<0.05.

M (QL, QU), Median (Lower Quartile, Upper Quartile); N (M/F), Number of participants (Male/Female); DM, Diabetes Mellitus; BMI, Body Mass Index; WBC, White Blood Cell count; NE, Neutrophil count; LY, Lymphocyte count; MC, Monocyte count; RBC, Red Blood Cell count; Hb, Hemoglobin; PLT, Platelet count; PDW, Platelet Distribution Width; MPV, Mean Platelet Volume; MCV, Mean Corpuscular Volume; MCH, Mean Corpuscular Hemoglobin; FPG, Fasting Plasma Glucose; HbA1c, Hemoglobin A1c; TG, Triglycerides; TC, Total Cholesterol; HDL-C, High-Density Lipoprotein Cholesterol; LDL-C, Low-Density Lipoprotein Cholesterol; AST, Aspartate Aminotransferase; ALT, Alanine Aminotransferase; ALB, Albumin; ALP, Alkaline Phosphatase; NLR, Neutrophil-to-Lymphocyte Ratio; SIRI, Systemic Inflammation Response Index; PLR, Platelet-to-Lymphocyte Ratio; PHR, Platelet-to-Hemoglobin Ratio; GGT, Gamma-Glutamyl Transferase; Scr, Serum Creatinine; SUA, Serum Uric Acid; LVEF, Left Ventricular Ejection Fraction.

### 3.3 Logistic regression analysis of factors influencing liver fibrosis in T2DM patients with MAFLD

Logistic regression analysis, using the degree of liver fibrosis as the dependent variable and statistically significant indicators from **Table 2** (excluding those with multicollinearity) as independent variables, revealed that NLR, SIRI, PLR, and PHR were significant factors influencing liver fibrosis in T2DM patients with MAFLD (**Table 3**).

**Table 3:** Logistic regression analysis of influencing factors for T2DM complicated with MAFLD liver fibrosis.

Variable	β	SE	Wald χ^2^	OR(95%CI)	P
Suspected liver fibrosis					
**NLR**	0.578	0.172	11.313	1.783(1.273-2.497)	0.001
**SIRI**	1.084	0.299	13.165	2.957(1.646-5.312)	<0.001
**PLR**	-0.006	0.004	3.211	0.994(0.987-1.001)	0.073
**PHR**	-0.018	0.003	32.932	0.983(0.977-0.989)	<0.001
Advanced stage of liver fibrosis					
**NLR**	1.137	0.295	14.804	3.117(1.747-5.563)	<0.001
**SIRI**	2.303	0.524	19.285	10.002(3.579-27.953)	<0.001
**PLR**	-0.031	0.011	8.341	0.970(0.950-0.990)	0.004
**PHR**	-0.032	0.009	13.489	0.969(0.953-0.985)	<0.001

β, Regression coefficient; SE, Standard Error; Wald χ2, Wald Chi-square statistic; OR (95% CI), Odds Ratio (95% Confidence Interval); P, P-value; NLR, Neutrophil-to-Lymphocyte Ratio; SIRI, Systemic Inflammation Response Index; PLR, Platelet-to-Lymphocyte Ratio; PHR, Platelet-to-Hemoglobin Ratio; T2DM, Type 2 Diabetes Mellitus; MAFLD, Metabolic-Associated Fatty Liver Disease.

### 3.4 ROC curve analysis of NLR, SIRI, PLR, and PHR in predicting liver fibrosis in T2DM patients with MAFLD

**Figure 1:**
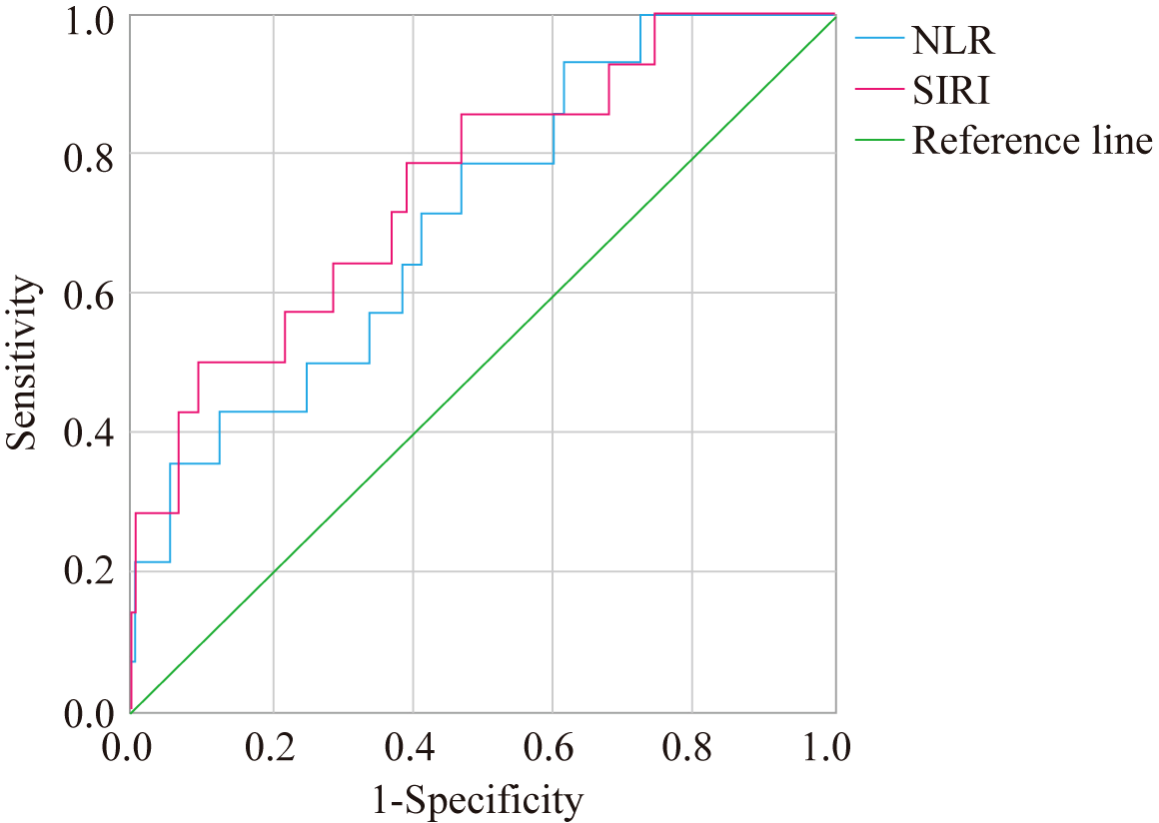
ROC curve analysis of NLR and SIRI in predicting liver fibrosis.

**Figure 2:**
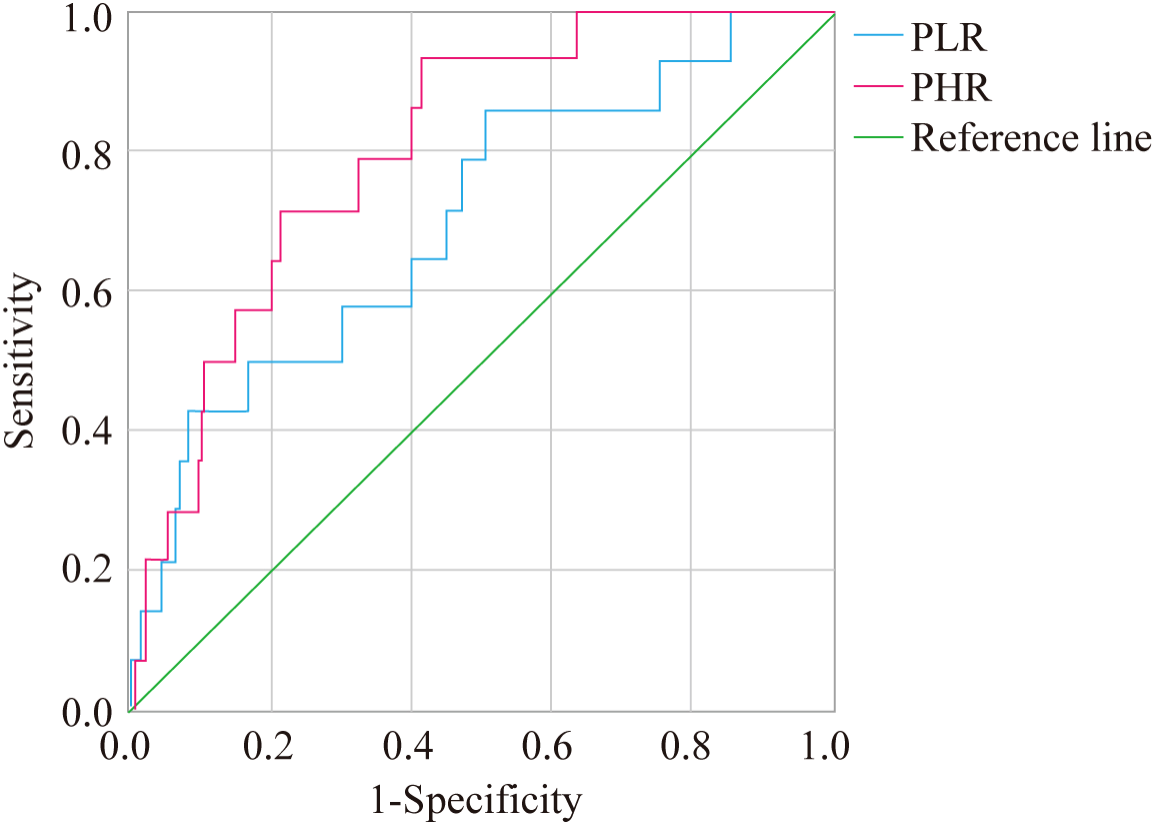
ROC curve analysis of PLR and PHR in predicting liver fibrosis.

ROC curve analysis showed that the AUC for NLR, SIRI, PLR, and PHR in predicting liver fibrosis in T2DM patients with MAFLD was 0.712, 0.757, 0.703, and 0.806, respectively. The sensitivity values for NLR, SIRI, PLR, and PHR were 0.929, 0.500, 0.857, and 0.929, respectively, while the specificity values were 0.388, 0.902, 0.497, and 0.593, respectively. The optimal cutoff values for NLR, SIRI, PLR, and PHR were 1.573, 1.465, 110.819, and 185.379, respectively (**Figures 1**, **2**).

## 4 Discussion

MAFLD is a common cause of chronic liver injury worldwide, with liver metabolic dysregulation playing a central role in the development of various metabolic diseases. The interplay between glucose and lipid metabolism significantly impacts insulin resistance (IR), a condition prevalent in most patients with T2DM. IR is a key factor in the progression of steatosis and non-alcoholic steatohepatitis, leading to dysfunction in hepatic adipose tissue. This dysfunction affects the production and secretion of adipokines and inflammatory cytokines, contributing to further liver damage (24). Our findings align with this mechanism, as the Experimental group exhibited elevated FPG, HbA1c, TG, and LDL-C—hallmarks of insulin resistance and dyslipidemia—which collectively drive hepatic inflammation and fibrogenesis.

In this study, we observed a lower prevalence of MAFLD in females compared to males, though females exhibited a higher proportion of liver fibrosis progression, especially in older age groups. This could be attributed to the decrease in estrogen levels post-menopause, which plays a crucial role in regulating mood, homeostasis, fat distribution and function, inflammatory responses, and IR (25). Estrogen deficiency exacerbates hepatic lipotoxicity and mitochondrial dysfunction, creating a pro-inflammatory microenvironment that favors fibrogenesis (26). The reduction in estrogen after menopause disrupts internal balance, promoting the development of MAFLD and increasing the likelihood of liver fibrosis in females. Additionally, the Experimental group had a significantly higher BMI compared to the Control group, reinforcing the association between high BMI and the development of MAFLD (27). Obesity-induced adipose tissue hypoxia and macrophage polarization toward a pro-inflammatory phenotype further amplify systemic inflammation, contributing to liver injury and fibrosis (28).

A national cross-sectional study (29) reported prevalences of steatosis, severe steatosis, advanced fibrosis, and cirrhosis at 44.39%, 10.57%, 2.85%, and 0.87%, respectively. In our study, 3.7% of participants had liver fibrosis, which aligns with these previous findings. The subgroups with progressive liver fibrosis in this study showed elevated levels of the NLR and the SIRI. Both NLR and SIRI are inflammatory markers calculated using NE, LY, and MC. Elevated NLR reflects a systemic imbalance between innate immune activation (neutrophilia) and adaptive immune suppression (lymphopenia), which is increasingly recognized as a hallmark of progressive liver fibrosis in MAFLD (30). In our cohort, the advanced fibrosis subgroup demonstrated a stepwise increase in NLR (1.573 cutoff), supporting its role as a sensitive marker of fibrotic progression. In the inflammatory stress response, margination and redistribution are key factors contributing to the decrease in LY in peripheral venous blood (31). NE plays a significant role in the innate immune response, including the release of inflammatory factors, mediators, and phagocytosis of dead or necrotic cells (18). Neutrophils exacerbate liver fibrosis by secreting MMPs and ROS, which directly damage hepatocytes and activate hepatic stellate cells. This process is amplified by bidirectional interactions between neutrophils and HSCs, as demonstrated in models of steatohepatitis and gut microbiota-driven inflammation (32, 33). This is consistent with our observation of elevated AST and ALT in fibrosis subgroups, indicative of ongoing hepatocyte injury.

SIRI, which incorporates the change in MC, emphasizes the importance of MC in clearing infections, promoting tissue repair, and supporting immune defense. However, the aggregation of MCs can exacerbate inflammation and lead to degenerative diseases (34). Recent evidence highlights that monocytes infiltrate the liver and differentiate into pro-fibrotic macrophages, secreting TGF-β and PDGF to stimulate collagen synthesis (35). In our study, the advanced fibrosis subgroup exhibited significantly higher SIRI (1.465 cutoff) compared to non-fibrosis subgroups, underscoring its specificity in reflecting monocyte-driven fibrotic processes.

Our study confirms that NLR and SIRI are influential factors in liver fibrosis progression in T2DM patients with MAFLD. Additionally, the decreases in PLR and PHR suggest an aggravated inflammatory state, which could promote the progression of liver fibrosis, showing a negative correlation with MAFLD-related fibrosis. Low PLR indicates thrombocytopenia relative to lymphopenia, reflecting both impaired liver synthetic function and heightened inflammation. Platelets themselves release pro-fibrotic mediators like serotonin and TGF-β, linking thrombocytopenia to reduced capacity for fibrosis modulation (36). This aligns with our findings of reduced PLT and PLR in advanced fibrosis subgroups. Reduced high-density lipoprotein cholesterol (HDL-C) is a well-established marker for increased risk of decompensation in chronic liver diseases, and it could effectively complement existing prognostic scoring systems (37). HDL-C exerts anti-inflammatory effects by inhibiting endothelial adhesion molecule expression and neutralizing oxidized lipids (38); its reduction in MAFLD exacerbates oxidative stress and hepatocyte apoptosis (39). The significant decrease in PHR (185.379 cutoff) in fibrosis subgroups highlights the dual impact of dyslipidemia and platelet dysfunction in fibrosis progression.

Logistic regression analysis identified PLR and PHR as significant factors influencing liver fibrosis in T2DM patients with MAFLD. Furthermore, ROC curve analysis demonstrated the predictive value of NLR, SIRI, PLR, and PHR, with NLR and PHR showing higher sensitivity (0.929 and 0.929, respectively), while SIRI exhibited greater specificity (0.902) in predicting liver fibrosis. These findings align with a 2023 study validating NLR and SIRI as robust predictors of fibrosis in MAFLD, with AUCs comparable to traditional biomarkers like FIB-4 (40). The superior AUC of PHR (0.806) in our study suggests that integrating lipid and platelet indices enhances fibrosis prediction, potentially due to their dual reflection of metabolic dysregulation and inflammatory burden.

One limitation of this study is its single-center design, which may introduce potential biases due to the influence of local dietary habits, lifestyle patterns, and genetic factors. Furthermore, the impact of medication use was not accounted for. To address these limitations, future research should include data from diverse geographical regions to improve the generalizability and applicability of the findings.

In summary, NLR, SIRI, PLR, and PHR are significant factors influencing liver fibrosis in T2DM patients with MAFLD. These inflammatory markers hold potential as valuable diagnostic tools to assist in identifying liver fibrosis in this specific patient population.

## Data Availability

The original contributions presented in the study are included in the article/Supplementary Material. Further inquiries can be directed to the corresponding author.
